# COVID 19 repercussions in ophthalmology: a narrative review

**DOI:** 10.1590/1516-3180.2021.0113.R1.0504221

**Published:** 2021-06-14

**Authors:** Thiago Gonçalves dos Santos Martins, Diogo Gonçalves dos Santos Martins, Thomaz Gonçalves dos Santos Martins, Paula Marinho, Paulo Schor

**Affiliations:** I MD, MSc. Doctoral Student, Universidade Federal de São Paulo (UNIFESP), São Paulo (SP), Brazil; Research Fellow, Ludwig-Maximilians-Universität (LMU), Munich Germany; and Doctoral Student, University of Coimbra, Coimbra, Portugal; II MD. Ophthalmologist, Hospital Central da Aeronáutica, Rio de Janeiro (RJ), Brazil.; III MD. Ophthalmologist, Hospital da Piedade, Rio de Janeiro (RJ), Brazil; IV MD. Doctoral Student, Universidade Federal de São Paulo (UNIFESP), São Paulo (SP), Brazil.; V MD, MSc, PhD. Professor, Department of Ophthalmology, Universidade Federal de São Paulo (UNIFESP), São Paulo (SP), Brazil.

**Keywords:** COVID-19, Ophthalmology, Coronavirus, Ophthalmologists, SARS-CoV-2, Coronavirus disease 2019, Severe acute respiratory syndrome coronavirus 2, Corona viruses

## Abstract

**BACKGROUND:**

The new coronavirus of 2019 (COVID-19) caused by severe acute respiratory syndrome coronavirus 2 (SARS-CoV-2) has spread globally and has repercussions within ophthalmological care. It has caused ocular manifestations in some patients, which can spread through eye secretions.

**OBJECTIVES:**

The purpose of this review was to summarize the currently available evidence on COVID-19 with regard to its implications for ophthalmology.

**DESIGN AND SETTING:**

Narrative review developed by a research group at Universidade Federal de São Paulo (UNIFESP), São Paulo (SP), Brazil, and at Ludwig-Maximilians-Universität, Munich, Germany.

**METHODS:**

We searched the literature on the repercussions of COVID-19 within ophthalmological care, using the MEDLINE and LILACS databases, with the keywords “COVID-19”, “ophthalmology” and “coronavirus”, from January 1, 2020, to March 27, 2021. Clinical trials, meta-analysis, randomized controlled trials, reviews and systematic reviews were identified.

**RESULTS:**

We retrieved 884 references, of which 42 were considered eligible for intensive review and critical analysis. Most of the studies selected reported the evidence regarding COVID-19 and its implications for ophthalmology.

**CONCLUSIONS:**

Knowledge of eye symptoms and ocular transmission of the virus remains incomplete. New clinical trials with larger numbers of patients may answer these questions in the future. Moreover, positively, implementation of innovative changes in medicine such as telemedicine and artificial intelligence may assist in diagnosing eye diseases and in training and education for students.

## INTRODUCTION

The outbreak of the new coronavirus (COVID-19) that started in Wuhan, China, has spread all over the world and has had a great impact on eye care.^1^ It is a ribonucleic acid (RNA) virus, called severe acute respiratory syndrome coronavirus 2 (SARS-CoV-2), which may have ocular manifestations in some patients.^2^

This is a single-stranded RNA virus with a genome of about 30 kb in length. The RNA genome encodes its proteins. The proteins are spike protein S, membrane protein M, envelope protein E and nucleocapsid protein N. Protein S is responsible for attachment to host receptors; protein M helps to shape virus particles and their binding to the nucleocapsid; protein E acts in the assembly of particle release; and protein N acts on genome binding and replication.^3^

The new coronavirus shows 96% genetic similarity to the bat-type coronavirus SARS BatCovRaTG13, and its spike surface protein (S) binds to angiotensin-converting enzyme 2 (ACE2) on the cell surface. ACE2 expression can be found in respiratory, intestinal, renal, cardiac and immune cells. Its main transmission routes are through respiratory droplets, fomites and fecal-oral routes. Some patients have had an episode of conjunctivitis before pneumonia, thus raising the hypothesis that the ocular mucosa is a possible transmission route for SARS-CoV-2, since the cornea and conjunctiva show expression of the ACE2 receptor, which is responsible for entry of the virus cells.^4^ Presence of the ACE2 infection receptor in the aqueous humor of humans has also been described.^5^ ACE2 is a crucial receptor for SARS-CoV-2 in vivo: in an experiment on mice, an injection of SARS-CoV spike worsened acute lung failure in vivo, which was then attenuated by blocking the renin-angiotensin pathway.^6,7^

## OBJECTIVE

The objective of this narrative review was to summarize the currently available evidence on COVID-19 with regard to its implications for ophthalmology.

## METHODS

We conducted a review of the literature considering the period from January 1, 2020, to March 27, 2021. We used the MEDLINE database (via PubMed) and LILACS (via Virtual Health Library) to identify relevant articles on the repercussions of COVID-19 within ophthalmological care, without restrictions on languages. Different combinations of keywords and MeSH terms were used as search strategies in order to ensure a broad search strategy: “ophthalmology”, “COVID-19” and “coronavirus”. The titles and abstracts of citations identified through these search strategies were screened for eligibility. We selected the main articles that reported on ophthalmological manifestations of coronavirus and how the pandemic was impacting ophthalmological care and education. We also selected articles that presented alternative solutions for eye care, such as the use of telemedicine and artificial intelligence. The details of the search strategy are shown in **Table 1**.

**Table 1:** Details of the search strategy

Database	Search strategies	Papers found
MEDLINE (via PubMed)	#1- (“ophthalmology”) AND (“coronavirus”)	872
	#2-(“COVID-19” [Mesh Terms) AND (“ophthalmology” [MeSH Terms])	220
LILACS (via Biblioteca Virtual em Saúde, BVS)	#1- (“ophthalmology”) AND (“coronavirus”)	300
	#2- (“COVID-19” [Mesh Terms) AND (“ophthalmology” [MeSH Terms])	7

## RESULTS

**Figure 1:**
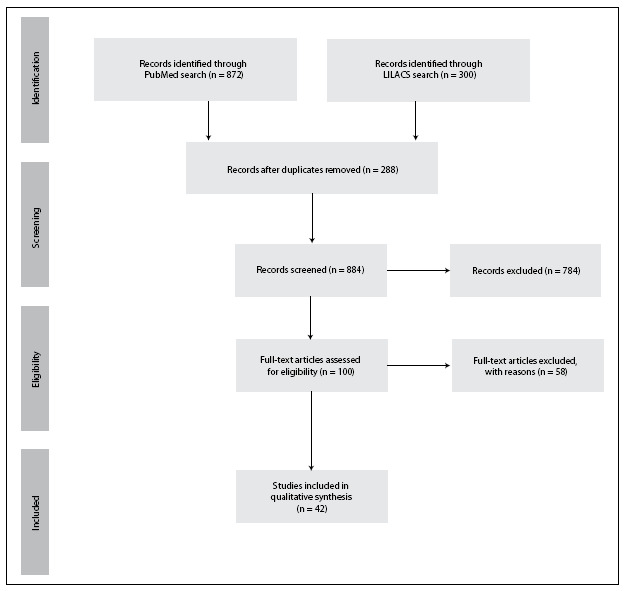
Flow diagram of the study selection process.

From the search in the databases, two clinical trials, 11 meta-analyses, one randomized controlled trial, 158 reviews and 27 systematic reviews were identified. After screening the titles and abstracts, removing duplicates and screening the citations, 42 studies were considered eligible for critical analysis. The article selection process is detailed in **Figure 1**.

### Ophthalmological manifestations

The main symptom reported by infected patients was viral conjunctivitis (hyperemia, eye pain, photophobia and tearing), which lasted for 2 to 24 days (average of 6 days). The incubation period for this viral infection is 2 to 14 days.^8^

In a study conducted among a total of 535 patients with COVID-19, 5.0% had conjunctival congestion, 29.6% had conjunctival discharge, 22.2% had watery eyes and 18.5% had ocular pain. Eye symptoms were usually present in patients with more severe symptoms of the disease. In that study, no viral nucleic acid was detected in the conjunctival swabs taken from the patients.^9^ In another study on 1,099 patients, presence of conjunctival congestion was detected in 0.8% of the patients. Conjunctivitis in these patients usually has follicles and mucoid secretion.^10,11^ Children can be affected by conjunctivitis, as well as adults, and they may present a picture of eyelid dermatitis.^12^ The viral load of conjunctival sac secretions in patients with COVID-19 is relatively low and is usually proportional to the severity of the disease.^13^

Xia et al. reported that SARS-CoV-2 existed only in the conjunctival and lacrimal secretions of a patient with conjunctivitis and that patients without ocular manifestations would not be a source of infection via this transmission route.^14^ However, another study demonstrated the presence of the virus in the eye secretions of patients without conjunctivitis.^15^ Therefore, ophthalmological examinations need to be carried out in a ventilated place and all possible measures should be taken to avoid cross-infection among ophthalmic patients. Patients should be advised not to touch their eyes because of the risk of contamination by the virus. The recommended treatment for viral conjunctivitis is supportive, and most cases are self-limiting. However, some recommendations for decreasing transmission rates should be followed, such as frequent hand washing and avoidance of touching one’s eyes.

The coronavirus epidemic has changed the criteria for corneal donation in some countries. In these, exclusion of suspected or confirmed cases has been started.^16^ Coronavirus patients may present with hyperreflective lesions at the level of ganglion cells and in the inner plexiform layer. This condition may be associated with hemorrhages and cottony exudates.^17^ Cases of changes in ocular motility after coronavirus infection have been described.^18^ Clinical findings such as conjunctivitis, retinitis, anterior uveitis and optic neuritis have been recognized in animal models. Thus, awareness of these possible manifestations in humans is needed.^19^

### Ophthalmological services

Because ophthalmological care is administered close to patients’ faces, ophthalmologists are exposed to tears and eye discharges. These medical specialists are therefore at high risk of contracting COVID-19. Protective measures for the mouth, nose and eyes need to be implemented, with use of personal protective equipment (N95, FFP2 or FFP3 masks, goggles and face shields).

Reduction of the number of patients scheduled should be sought, along with greater distance (at least 1.5 meters) between patients in the waiting room. Online prescription programs for patients can be implemented. Patients should have their temperature measured before entering the doctor’s office and must wear a mask for the entire period of stay in the doctor’s office. If the patient’s temperature is above 37.5 °C, the appointment should be postponed unless it is an eye emergency.

The examination room needs to be well ventilated, and the ophthalmic devices should be disinfected immediately after use, with 0.1% sodium hypochlorite or 70% ethanol for at least one minute before and after examination of the patient. Use of a non-contact tonometer should be avoided because of the micro-aerosols that are generated. The tips of the Goldman tonometer need to be disinfected.^20,21^

Examinations using direct ophthalmoscopy should be avoided due to the proximity to the patient. This can be replaced by retinography, which allows greater distancing from the patient. Acrylic shields need to be installed in the slit lamp, so as to reduce contact with aerosols generated by patients. Ophthalmological examinations should be conducted as soon as possible. Elective procedures should be postponed during this period. Emergency surgery for infected patients should be performed preferably in operating rooms with negative pressure. Moreover, surgery with general anesthesia should be avoided, given that intubation can generate an aerosol.

So far, there is no evidence to contraindicate use of contact lenses by patients. However, because it is known that the virus can be isolated in tears and conjunctiva, it is advisable to avoid adaptation to contact lenses during this period. The main objectives of these measures are to minimize cross-infection between employees and patients, and to ensure a safe working environment.

### Telemedicine

The training of ophthalmology students has been greatly affected during the pandemic period, since most appointments and elective surgeries have been canceled.^22^ Telemedicine with use of distance training has become an option for training clinical staff during this period. The teleguidance for ophthalmological patients has also been used, with the aims of correctly directing emergencies to hospitals and preventing patients from unnecessarily looking for hospitals, which might increase their risk of contamination. During this period, use of training models and simulators has proven to be useful in training these students for surgery and eye examination.^23,24,25^

Patients can also be assisted through teleophthalmology, which can be improved with appropriate training for healthcare professionals. Quality images can be captured and sent to ophthalmologists in other locations. In special pandemic situations, some images can even be captured by patients using smartphones.^26,27^ The COVID-19 pandemic has transformed some leading telehealth platforms, which have reported that virtual visits by patients have increased by between 257% and 700%.^28^

For evaluation of macular diseases, there are applications and devices to monitor the central 10 degrees of the field of view, such as the hyperacuity perimeter ForSee device (Notal Vision).^29^ There are already smartphones capable of capturing images of the retina, but they have not yet been designed for patients to use at home.^30^

During this period of global health emergency, rapid communication and international collaboration are essential, in order to achieve better outcomes from the pandemic, and telemedicine has collaborated in this process. Teleophthalmology plays an important role in rural areas and in places with few specialists, but during this pandemic it has become useful even in areas with enough ophthalmologists, in order to promote social isolation and reduce the rate of virus transmission.

### Artificial intelligence

In this scenario of difficult access to eye care, artificial intelligence seems to be an alternative for facilitating the diagnosis and monitoring eye diseases. Through the development of artificial intelligence, it has become possible to develop algorithms that are able to identify lesions in ophthalmic examinations without human intervention. This enables analysis of the large amounts of data that are generated, in an automatically supervised, semi-supervised or unsupervised manner. Development of algorithms with real-time cloud database analysis may be useful for controlling and monitoring eye diseases. Thus, incorporation of machine-learning technology in ophthalmology can improve medical care for the population in regions with limited medical resources, thereby reducing some social inequalities that already existed before the coronavirus epidemic and became more evident.

Development of algorithms in smartphone applications helps in identifying contacts with contaminated people (contact tracing). These algorithms cross-reference information about patients’ locations and symptoms. They are responsible for notifying people who have been in contact with infected individuals regarding the need for quarantine, so as to reduce the risk of spreading contamination across society. Artificial intelligence is providing new ways to enable contact tracking, through using Bluetooth to track nearby phones, keep records of these contacts and alert people to others with whom they have been in contact. An individual can test positive and then the algorithms start a cascade of notifications from all recent contacts. Alternatively, an individual may be notified that he was in the vicinity of another anonymous person who tested positive. This big data can be used by public healthcare systems. Some problems still need to be overcome, such as maintaining data confidentiality and the fact that not all people have smartphones and internet access.^31,32,33,34,35^ In China, the Alipay Health app on Alipay indicates the possibility of getting around a city, based on three categories: green (without restrictions), yellow (7-day quarantine) and red (14-day quarantine).^36^ An application developed in South Korea warns people by text message if they were close to people diagnosed with COVID-19.^37^ In Italy, the application “Immuni” combines clinical information with the possible contacts of infected people.^38^

The WellAI COVID-19 app uses deep neural networks to learn from a big dataset about COVID-19 and to summarize existing knowledge. These algorithms are based on unsupervised learning.^39^ SciSight is an algorithm that uses artificial intelligence to explore associations between concepts that appear in the COVID-19 dataset. It is available at https://SciSight.apps.allenai.org/.^40^ These algorithms and large databases (big data) are valuable within research and development relating to medical knowledge. Artificial intelligence tools are expected to accelerate development of the diagnosis, treatment and prevention of COVID-19, at a time when humanity needs rapid responses in order to minimize the damage of the pandemic.

## DISCUSSION

The COVID 19 epidemic has had a major impact on the volume of eye care. The reduction in the volume of care that has occurred has hindered the teaching of ophthalmology residents and the follow-up of several ophthalmic diseases. Use of technology in telemedicine and development of artificial intelligence algorithms can reduce the impact of COVID-19 on the care of ophthalmic patients.^41^

The policies of lockdown and social distancing have hindered the ophthalmological follow-up of many patients. Many elective eye surgeries have been canceled and treatments for many patients have been delayed.^42^ During the period of greatest social isolation, some hospitals even reported a reduction of 63% in the number of ophthalmological consultations and 67% in the number of surgeries. On the other hand, they reported an increase of 1800% in the number of telemedicine consultations.^42^ The impact on the population’s eye health can only be clarified through future studies that may document what this loss of ophthalmological monitoring may have caused.

The period of social isolation and the large number of deaths caused by the coronavirus pandemic have caused a major change to the mental health of ophthalmologists. The numbers of psychiatric complaints among them, such as depression, anxiety and insomnia, have increased.^43^ Since ophthalmologists perform examinations very close to patients’ faces, they are at high risk of contamination. In a survey conducted at Moorfields Eye Hospital, 80% of the ophthalmologists were found to be at high risk of contamination by coronavirus.^44^ The main concerns were the lack of adequate protective masks and the risk of contaminating family members. Many patients have reported fear of loss of vision during the pandemic due to loss of eye care.^45^ Thus, not only is patients’ health impaired through social isolation, but also ophthalmologists’ health is impaired through the risk of contamination and mental health problems.

The new forms of assistance by means of telemedicine and with the use of artificial intelligence have limitations in countries that do not have an adequate internet infrastructure and where the population does not have access to this technology.^46^

However, a study has shown that most patients like this new form of telemedicine care. Thus, this new service can continue to be used even after the end of the coronavirus pandemic.^47^ Telemedicine consultations can reduce the number of unnecessary consultations with ophthalmologists. This reduces the risk of infection, reduces the cost of healthcare and increases the degree of patient satisfaction.

### Future perspectives

Because COVID-19 is a new virus, further studies will be needed to characterize the main ophthalmological symptoms and whether the ocular route can provide a gateway for the virus, as a primary infection site. Through new studies, more can be learned about the pathophysiology of this disease and a better theoretical basis for better orientation of the population can be established. Development of new artificial intelligence algorithms and devices for capturing ophthalmological images, which can be used by patients themselves, can facilitate the monitoring of ophthalmic diseases in areas with few specialists or in situations that require social isolation, such as epidemics.^48,49,50,51^ Academic medical centers should take this opportunity to modify their curricula, including training in data science, computing, virtual reality and telemedicine. The expectation is that, with increasing levels of vaccination in the population, eye care and the number of surgeries will be able to return to normal within a short time.^52^

### Strengths and limitations

The articles included in this review generated heterogeneous data because of the diversity in the design of the studies (clinical trials, meta-analysis, randomized controlled trials, reviews and systematic reviews). The main limitation of this review was the lack of tools for methodological assessment of the reviews. This narrative review does not provide quantitative answers to specific questions about the ophthalmic manifestations of coronavirus. The selection of studies and the interpretation of information may have been influenced by the authors’ subjectivity.

## CONCLUSION

Since 2002, coronaviruses have seemed to pose a continuous threat to humanity, such that there is a need to always be on the lookout for new outbreaks. Knowledge of eye symptoms and ocular transmission of the virus remains incomplete. New clinical trials with larger numbers of patients may answer these questions in the future. Moreover, positively, implementation of innovative changes in medicine such as telemedicine and artificial intelligence may assist in diagnosing eye diseases and in training and education for students.
